# Proximal tubule hypertrophy and hyperfunction: a novel pathophysiological feature in disease states

**DOI:** 10.1093/ckj/sfae195

**Published:** 2024-06-25

**Authors:** Mehmet Kanbay, Sidar Copur, Mustafa Guldan, Lasin Ozbek, Alper Hatipoglu, Adrian Covic, Francesca Mallamaci, Carmine Zoccali

**Affiliations:** Department of Internal Medicine, Division of Nephrology, Koç University School of Medicine, Istanbul, Turkey; Department of Medicine, Koç University School of Medicine, Istanbul, Turkey; Department of Internal Medicine, Division of Internal Medicine, Koç University School of Medicine, Istanbul, Turkey; Department of Internal Medicine, Division of Internal Medicine, Koç University School of Medicine, Istanbul, Turkey; Department of Internal Medicine, Division of Internal Medicine, Koç University School of Medicine, Istanbul, Turkey; Nephrology, Dialysis and Transplantation, University Grigore T Popa, Iasi, Romania; Nephrology, Dialysis and Transplantation Unit, Grande Ospedale Metropolitano, Reggio Calabria, Italy; CNR-IFC, Research Unit of Clinical Epidemiology and Physiopathology of Renal Diseases and Hypertension, Institute of Clinical Physiology, Reggio Calabria, Italy; Renal Research Institute, New York, NY, USA; Institute of Molecular Biology and Genetics, Ariano Irpino, Italy; Associazione Ipertensione Nefrologia Trapianto Renale, Grande Ospedale Metropolitano, c/o Nefrologia, Reggio Calabria, Italy

**Keywords:** chronic kidney disease, diabetes mellitus, proximal tubules, sodium–glucose cotransporter 2 (SGLT2), tubular hypertrophy

## Abstract

The role of proximal tubules (PTs), a major component of the renal tubular structure in the renal cortex, has been examined extensively. Along with its physiological role in the reabsorption of various molecules, including electrolytes, amino acids and monosaccharides, transcellular transport of different hormones and regulation of homeostasis, pathological events affecting PTs may underlie multiple disease states. PT hypertrophy or a hyperfunctioning state, despite being a compensatory mechanism at first in response to various stimuli or alterations at tubular transport proteins, have been shown to be critical pathophysiological events leading to multiple disorders, including diabetes mellitus, obesity, metabolic syndrome and congestive heart failure. Moreover, pharmacotherapeutic agents have primarily targeted PTs, including sodium–glucose cotransporter 2, urate transporters and carbonic anhydrase enzymes. In this narrative review, we focus on the physiological role of PTs in healthy states and the current understanding of the PT pathologies leading to disease states and potential therapeutic targets.

## INTRODUCTION

The proximal tubules (PTs), predominantly located in the renal cortex, are vital components of the nephron, starting from the Bowman's capsule and extending to the initial segment of the loop of Henle [1]. These tubules have a high capacity for both active and passive reabsorption, making them the primary site for the reabsorption of most substances, excluding magnesium, which is predominantly reabsorbed in the thick segment of the ascending limb of the loop of Henle [2]. Sodium–glucose cotransporter 2 (SGLT2) and glucose transporter 2 (GLUT2) handle ≈90% of glucose reabsorption in the first segment, with SGLT1 and GLUT1 managing the rest in subsequent segments [3, 4]. Hyperfunctioning of PTs refers to an increased rate of these physiological processes, resulting in augmented glucose, sodium and amino acid reabsorption from the glomerular filtrate [5], which in some circumstances may contribute to electrolyte imbalances, alterations in acid–base balance and other metabolic disturbances. This can occur secondary to hormonal imbalances, renal disorders or pharmacological influences, ultimately impacting renal function and overall physiological homeostasis [5]. In contrast to glomerular function, the assessment of PT function is a much more challenging issue. requiring simultaneous measurement of serum and ultrafiltrate levels of various molecules or drugs. A few examples of PT function assessment tools include tubular maximum phosphate reabsorption capacity, urate reabsorption capacity and glomerular filtration rate (GFR) [6, 7].

On the other hand, PT hypertrophy refers to enlargement or expansion of the PT epithelial cells in the kidneys. This phenomenon typically occurs in response to various pathological conditions, including acute kidney injury (AKI), chronic kidney disease (CKD) and exposure to nephrotoxic substances. The mechanism underlying PT hypertrophy involves complex cellular processes to adapt to stress and restore renal function [8]. Nevertheless, there is no consensus on the definition or cut-off measurement values for PT hypertrophy.

PT function is intimately linked with the pathogenesis of diseases, from metabolic disorders like diabetes and obesity to cardiovascular diseases. Furthermore, various medications, including SGLT inhibitors, urate transporter inhibitors and acetazolamide, exert their effects through modulation of PT activity [9]. This review explores the multifaceted importance of PTs, emphasizing their role in disease pathogenesis, their impact in pharmacotherapy and the significance of preserving and reversing pathological changes within this fundamental renal structure.

## PT FUNCTION

**Figure 1:**
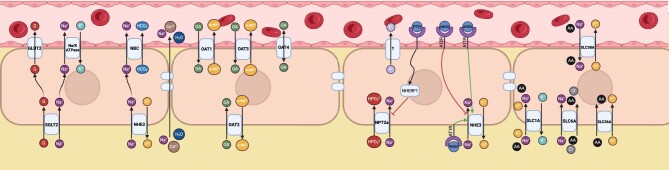
The functions of PTs in the reabsorption of various electrolytes through multiple transporters. NBC: sodium bicarbonate cotransporter; AQP: aquaporin; SLC: solute carrier protein; NPT: sodium–phosphate co-transporter; ENaC: epithelial sodium channel; NHERF1: sodium–hydrogen exchanger regulatory factor-1; G: glucose; P: phosphorus; HCO_3_: bicarbonate; Ca: calcium; OA: organic anion; H_2_O: water; ANG: angiotensin; V2R: vasopressin type II receptor.

The PT (Fig. 1) is a single layer of epithelial cells with microvilli on the luminal surface, giving it a brush border appearance. This increases the surface area for reabsorption. PTs are divided into two parts: pars convolute and pars recta. The S1 and S2 segments make up the pars convoluta and the S2 and S3 segments make up the pars recta [10]. Cells of the PTs, particularly those of the S1 segment, are rich in mitochondria [11]. PTs reabsorb filtered sodium, water, glucose, amino acids, phosphate, organic anions and cations, which are molecules that carry a charge and are involved in various physiological processes in the body. Organic anions include physiological compounds such as citrate, urate, oxalate, creatinine and drugs such as penicillin. Organic cations include neuromediators and neuromodulators such as dopamine, histamine and epinephrine and drugs such as cimetidine and metformin. By regulating the secretion and reabsorption of organic anions and cations, the PTs help to maintain the body's overall balance of ions and other important molecules. Additionally, PTs play a role in maintaining and regulating filtered macromolecule transportation via endocytosis.

## TRANSPORTATION THROUGH THE PTS

At the PTs, glucose reabsorption occurs in two steps. First, low-affinity SGLT2 takes up to 97% in the pars convoluta, and high-affinity SGLT1 takes the remainder in the pars recta [12, 13]. Sodium (Na)-dependent transporters generally reabsorb amino acids, but some transporters also depend on chlorine (Cl), potassium (K) and hydrogen (H) gradients [14]. Amino acids also have a similar transportation rationale. Low-affinity peptide transporter 1 (PEPT1) in the early PT recovers most of the di- and tripeptides, while high-affinity PEPT2 in the late PT reabsorbs the remainder [15], achieving complete reabsorption of amino acids. Furthermore, the kidney prevents any possible leakage of non-pathogenic proteins by reabsorbing them via megalin and cubilin, two endocytic receptors highly expressed in the renal PT. When these proteins are dysfunctional, it can lead to tubular proteinuria or can provide entry of toxic macromolecules into the circulation. Targeting these endocytic receptors could offer new opportunities for developing innovative treatment approaches for tubular proteinuria and prevention of macromolecular toxin accumulation within the body [16]. Potential therapeutic strategies involve modulating the activity of these endocytic receptors to improve protein reabsorption in the kidneys. Small molecules or peptides can regulate the function of megalin and cubilin, such as angiotensin II and transforming growth factor β (TGF-β) [17], and, in theory, gene therapy techniques can restore or upregulate the expression of these receptors.

Passage of ions in the PTs can occur para- or transcellularly. Thus claudin-2 forms a channel that controls the paracellular passage of small cations such as Na^+^ and K^+^ [18]. On the other hand, claudin-10a and claudin-17, both members of the claudin family of tight junction proteins, form channels that control the passage of anions across the nephron paracellularly [18]. The transcellular transportation of sodium is warranted by SGLT1 and 2, sodium–hydrogen exchanger 3 (NHE3 and the corresponding gene *SLC9A3*), sodium–phosphate co-transporter type 2 (NaPi2 and the corresponding gene *SLC34A3*), sodium amino acid transporters, sodium–potassium–adenosine triphosphatase (Na^+^/K^+^ ATPase) and sodium bicarbonate co-transporter [19]. Most of the ion transport from or to the nephron in PTs is coupled with sodium transportation. Finally, via organic acid transporters (OATs) and organic cation transporters (OCTs), the PTs play an important role in the maintenance and clearance of toxic products such as drugs and some metabolites (e.g. creatinine, uric acid, folate, indoxyl sulphate, hippuric acid) from the body [19].

## HORMONAL CONTROL OF PT FUNCTION

Parathyroid hormone (PTH) acts on PTs via the PTH1R receptor. This is a G protein–coupled receptor located on the cell membranes of PTs. Activation of PTH1R triggers the production of cyclic adenosine monophosphate (cAMP) from ATP by adenylate cyclase. The increase in cAMP acts as a second messenger, initiating a signalling cascade within the cell. This cascade commonly involves the activation of protein kinase A (PKA). PKA phosphorylates various target proteins within the proximal convoluted tubule cells, leading to several physiological responses, including increased reabsorption of calcium and decreased reabsorption of phosphate secondary to reduced expression of sodium–phosphate co-transporters on the apical surface of PT cells, which increases phosphate excretion. On the other hand, in the PTs, PTH stimulates the conversion of 25-hydroxyvitamin D into its active form, 1,25-dihydroxyvitamin D (calcitriol), which further increases calcium absorption from the gut [20, 21]. PTs also interfere with blood pressure control via the angiotensin II–regulated sodium transporters [22] such as the Na–H antiporter [23]. In physiological conditions, the effect of angiotensin II on the AT1 receptor is counterbalanced by the AT2 receptor [24, 25].

Insulin receptor substrate, Akt and the mammalian target of rapamycin (mTOR) complex serve as important mediators for insulin’s effect on the PTs. Insulin inhibits gluconeogenesis in the PTs [26], which impacts the availability of substrates like lactate, amino acids and glycerol for other metabolic pathways, possibly diverting them toward other processes such as protein synthesis or triglyceride formation. Notably, this hormone increases glucose reabsorption by acting on SGLT2, which can indirectly enhance sodium reuptake. Furthermore, it can influence the PT's OATs. These transporters are responsible for the uptake and excretion of a wide range of organic anions, including various drugs, toxins and endogenous metabolites. Insulin might also affect the activity of other transporters that work in tandem with OATs, such as those involved in the exchange or efflux of organic cations. By influencing cellular metabolism and energy availability, insulin may affect the energy-dependent processes that govern OAT function, including those transporters that work via secondary active transport mechanisms [27]. Overall, the effects of insulin on renal transport systems can be complex and influenced by various factors, including insulin sensitivity, the presence of other hormones and signalling molecules and the overall metabolic state of the individual. Also, insulin increases the expression and effectivity of OATs in the PT [28].

## THE ROLE OF PTS IN DISEASE STATES

PT hypertrophy and hyperfunction play significant roles in the pathogenesis and progression of CKD, especially in the context of inherited kidney disorders, nephron loss and diabetes-induced renal damage [5] (Table 1). PT hypertrophy is a compensatory mechanism in conditions of reduced nephron number or chronic hyperglycaemia [5]. As a result of the excessive glucose in the tubular lumen, the PT cells become overwhelmed and cannot effectively transfer all of the glucose to the peritubular capillaries. This leads to an accumulation of glucose within the cells, triggering a compensatory response in the form of hypertrophy [29]. This hypertrophy is the body's way of coping with the increased workload placed on the PT cells due to the glucose overload. This hypertrophy results in hyperreabsorption, hyperfiltration and increased production of reactive oxygen species, contributing to oxidative stress and angiotensinogen production [30]. The persistent increase in oxygen consumption by hypertrophied PTs may exacerbate CKD or ageing progression due to energy conservation concerns. Additionally, although compensatory renal hypertrophy involves various anatomical components of the nephron, the PT component is a primary driver for compensatory renal hypertrophy [31–33]. PT cell proliferation, rather than hypertrophy, represents an early response to hyperglycaemia and SGLT2 upregulation in diabetic kidney disease in mice [34].

**Table 1:** Comprehensive overview and research recommendations on PT dysfunction in cardiorenal disorders.

Disorders	Knowns	Unknowns	Future research recommendations
Pathophysiology	PT hypertrophy compensates for nephron loss or chronic hyperglycaemia, crucial in CKD progression under stressors like genetic disorders and diabetes-induced renal damage	The influence of genetic variations and polymorphisms on PT function, disease susceptibility and treatment response requires further elucidation.	Conduct longitudinal studies to assess how PT hypertrophy affects CKD progression and associated complications across diverse patient populations.
DM and hyperglycaemia	Hyperglycaemia enhances glucose and sodium reabsorption via SGLT2 and SGLT1. Intracellular glucose accumulation in PT cells leads to hypertrophy, hyperreabsorption, hyperfiltration and increased reactive oxygen species production. Diabetic nephropathy shows early structural changes and functional decline in PT cells. Observed in diabetes and diabetic nephropathy in animal models and clinical studies.	Variations in PT alterations across different stages of diabetic nephropathy and patient demographics. Comprehensive understanding of how PT dysfunction interacts with other pathophysiological mechanisms in diabetic kidney disease.	Explore novel therapeutic targets within PTs for diabetic nephropathy treatment. Investigate how PT alterations affect renal and cardiovascular outcomes in diabetic patients.
Obesity	Hypertrophy in response to obesity-induced metabolic and haemodynamic demands. Enhanced function via increased sodium and water reabsorption through SGLT1 and SGLT2, with pathophysiological mechanisms including lipid accumulation, activation of RAS and RhoA and aberrant hypoxic responses.	Mechanisms underlying the relationship between obesity and PT alterations, including pathways leading to hypertrophy and dysfunction. Long-term effects of PT hypertrophy on kidney function and disease progression in obese individuals.	Characterize the signalling pathways and molecular mechanisms driving PT alterations in obesity-related kidney dysfunction. Explore factors influencing the vulnerability of hypertrophic PT cells to hypoxia in obesity, identifying potential therapeutic targets.
HF	HF induces haemodynamic and neurohumoral changes that enhance Na^+^ and water reabsorption in PTs. Medications targeting PTs, such as SGLT2 inhibitors and proximal-acting diuretics, can alleviate volume overload in HF.	Mechanisms underlying PT alterations in response to HF-induced haemodynamic and neurohumoral changes. Molecular pathways and targets mediating PT hypertrophy with loop diuretic therapy. Strategies to optimize PT targeting for improved diuretic efficacy and HF outcomes.	Conduct longitudinal studies to assess the long-term effects of PT alterations on HF progression. Elucidate the role of IGF-1 signalling and other pathways in mediating PT hypertrophy induced by loop diuretic therapy. Conduct clinical trials to identify predictors of optimal response to SGLT2 inhibitors in HF patients.
Cardiac arrhythmia and SCD	SGLT2 inhibitors directly affect cardiomyocytes by inhibiting Na^+^/H^+^ exchanger activity and suppressing late Na^+^ currents. Meta-analysis of trials shows a reduced risk of SCD in HF patients treated with SGLT2 inhibitors, with varying effects in those with reduced ejection fraction.	Mechanisms of anti-arrhythmic effects of SGLT2 inhibitors via PT function. Influence of SGLT2 inhibitors on cardiac electrophysiology and arrhythmogenesis. Long-term effects of SGLT2 inhibitor therapy on arrhythmic outcomes in HF patients, including ventricular and atrial arrhythmias.	Conduct prospective trials to explore the long-term impacts of SGLT2 inhibitor therapy on arrhythmic outcomes in different ejection fraction subgroups. Investigate the direct effects of SGLT2 inhibitors on cardiac electrophysiology and arrhythmogenesis. Explore biomarkers associated with SGLT2 inhibitor therapy response in reducing SCD risk among HF patients.
Therapeutics	Drug delivery to PT cells involves overcoming anatomical barriers and utilizing receptor-mediated endocytosis, primarily via megalin and cubilin in the S1 segment.	PT dysfunction contributes to drug resistance or tolerance with certain therapies. Study molecular pathways for drug uptake and transport in PT cells to enhance drug delivery strategies.	Explore novel therapeutic targets within PTs for treating CKD and related disorders, targeting individual pathophysiological mechanisms in preclinical studies. Investigate drug delivery mechanisms to PT cells, focusing on proteins like megalin and cubilin, to enhance targeted therapy outcomes. Study the therapeutic potential of targeting RhoA activation and its effectors to mitigate obesity-induced kidney damage. Develop precision medicine approaches for renal disorders by integrating genetic, molecular and clinical data to tailor treatment strategies. Utilize advanced imaging techniques for real-time visualization of PT function to understand its role in health and disease. Conduct long-term clinical studies to assess the safety and efficacy of therapies for PT alterations in managing cardiorenal and metabolic disorders.
SGLT2 inhibitor	SGLT2 inhibitors inhibit PT sodium reabsorption and glucose uptake, leading to glycosuria and natriuresis. Emerged as significant therapeutic alternatives for type 2 DM and HF. Positive impacts on patient mortality, cardiovascular outcomes and the progression of kidney disease.	The precise mechanisms underlying the effects of SGLT2 inhibitors on the RAAS and sympathetic activity.	
Acetazolamide	Therapies targeting PT mechanisms, such as carbonic anhydrase inhibitors like acetazolamide, are effective in managing conditions such as HF.	Optimal dosing and administration schedules for therapies like acetazolamide in managing cardiorenal disorders, without inducing tolerance or metabolic acidosis, need to be established.	

**Figure 2:**
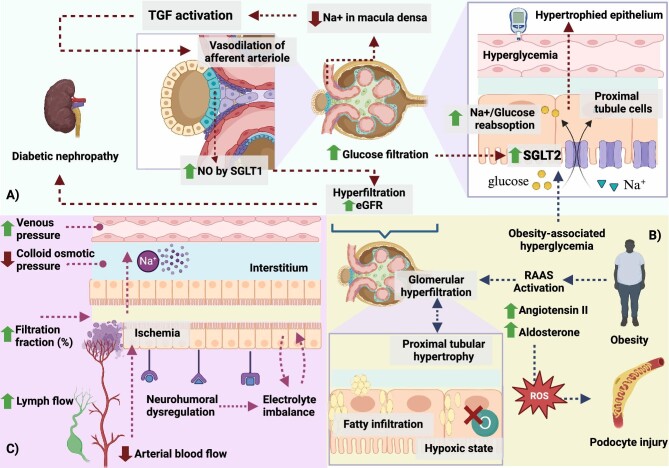
Pathophysiological alterations in the PT under conditions of diabetes, obesity and HF. (**A**) In diabetes, hyperglycaemia induces increased expression of SGLT2, leading to enhanced reabsorption of sodium and glucose. This disrupts tubuloglomerular feedback, causing vasodilation of the afferent arteriole and consequent hyperfiltration, compounded by nitric oxide generation by SGLT1 in the macula densa. Hyperfiltration ultimately contributes to diabetic nephropathy. (**B)** In obesity, hyperglycaemia-associated alterations mirror those observed in diabetes, including increased expression of SGLT2 and activation of tubuloglomerular feedback, resulting in hyperfiltration. Additionally, obesity-related factors such as RAAS activation contribute to glomerular hyperfiltration. Lipid droplets, indicative of triglyceride accumulation, were observed in PT cells in studies. Hypertrophic PT epithelium is also more vulnerable to a hypoxic state. (**C)** In heart failure, PT alterations play a significant role in the pathophysiology of the condition. HF induces haemodynamic and neurohumoral changes that increase Na^+^ and water reabsorption in the PTs. This is facilitated by an increased filtration fraction due to reduced renal blood flow, renal venous hypertension that alters hydrostatic pressure in the renal interstitium and peritubular capillaries and increased renal lymph flow, reducing colloid osmotic pressure in the renal interstitium. Consequently, diminished Na^+^ delivery to the distal nephron impacts the efficacy of diuretics and endogenous natriuretic peptides, exacerbating fluid retention and contributing to the progression of HF.

The hypertrophic response of PTs entails metabolic reprogramming to meet heightened energy demands. Concurrently, inflammatory and fibrotic reactions may occur, involving immune cell recruitment and cytokine release. Growth factors such as insulin-like growth factor 1 (IGF-1) and epidermal growth factor (EGF) regulate cell growth and differentiation. While PT hypertrophy aids renal adaptation and function, excessive or prolonged hypertrophy may exacerbate kidney disease progression [8]. Angiotensin II mediates hypertrophy of PT cells [35, 36]. Studies conducted on cultured PT epithelial cells show that hypertrophy in these cells, induced by hyperglycaemia, is primarily driven by a cell cycle–dependent mechanism. This finding highlights the molecular pathways involved in developing hypertrophy in response to hyperglycaemia, shedding light on potential therapeutic targets for diabetic nephropathy [37–39]. Understanding the mechanisms underlying PT hypertrophy can lead to developing targeted therapeutic strategies for CKD and related complications (Fig. 2).

The therapeutic benefits of SGLT2 inhibitors, which act by modifying PT function, have revealed their positive impact on patient mortality, cardiovascular outcomes and the progression of kidney disease [40]. Large-scale clinical trials demonstrated that SGLT2 inhibitors reduce the risk of kidney and heart disease in patients dealing with heart failure (HF), CKD, or type 2 diabetes at risk of atherosclerotic cardiovascular problems [9, 41]. A large meta-analysis documented that, independent of diabetes, SGLT2 inhibitors significantly reduce the risk of kidney disease progression by 37%, acute kidney injury by 23% and cardiovascular death or hospitalization for HF by 23% [9].

In subsequent subsections, we will delve into the current literature concerning PT hypertrophy and hyperfunction in various major diseases such as diabetes, obesity and heart diseases.

## DIABETES MELLITUS (DM) AND DIABETIC NEPHROPATHY AND PT ALTERATIONS

DM is one of the most comprehensively studied conditions concerning structural and functional shifts in PT [42–47]. Diabetes nephropathy is among the most investigated renal disorders in preclinical and clinical studies and thus is potentially useful in the understanding of PT hypertrophy and hyperfunctioning. The entry of glucose into PT cells is insulin independent and concentration dependent, with higher rates of glucose entry under hyperglycaemic conditions, which is mostly mediated by low-affinity, high-capacity SGLT2 transporter located in earlier segments of PTs and followed by high-affinity, low-capacity SGLT1 transporter located in later segments of PTs [48]. Such a physiological role is evident in studies illustrating none to minimal glucosuria in response to inactivating SGLT1 mutations in contrast to high rates of glucosuria in cases with inactivating SGLT2 mutations [49]. Increased expression of mRNA encoding for both SGLT1 and SGLT2 have been reported in animal models of streptozotocin-induced DM along with a similar outcome in obese diabetic Zucker rats compared with matched lean rats [50, 51]. A similar pattern of transporter expression has been observed in a preclinical study on human PT epithelial cell cultures [52].

Diabetic kidney growth and hyperfiltration are well-established clinical phenomena observed from the initial phases of diabetic kidney disease. PT growth under a hyperglycaemic environment involves an early period of tubular hyperplasia followed by tubular hypertrophy and involves complex pathophysiological pathways [53]. The understanding of such mechanisms is predominantly based upon preclinical studies involving cell cultures and animal models.

### Growth factors

In animal models of streptozotocin-induced DM, DNA synthesis peaks at day 2 while numerous growth factors, including IGF-1, vascular endothelial growth factor (VEGF), EGF, fibroblast growth factor (FGF) and platelet-derived growth factor (PDGF) have been shown to be upregulated and potentially involved in the early hyperplasia phase [45, 54]. Such growth factors, along with their downstream molecules, including the PI3K–Akt pathways and adenosine monophosphate–activated protein kinase (AMPK) pathway, are key signals inducing hyperplasia of tubular cells.

### Ornithine decarboxylase (ODC)

Ornithine decarboxylase (ODC) is a key rate-limiting enzyme in polyamine synthesis by decarboxylating ornithine into putrescine. Polyamines are crucial biomolecules for cellular growth and differentiation and overactivity of ODC has been implicated in tumour growth as well [55]. Overactivation of ODC has been linked to PT hyperplasia and hypertrophy in preclinical studies along with reversal of such conditions with ODC inhibition by difluoromethylornithine [55–57]. Both mTOR and protein kinase C pathways are involved in activating the ODC enzyme.

### TGF-β

TGF-β has been implicated in the switch from hyperplasia to hypertrophy by inducing G1 cell cycle arrest mediated via upregulation of cyclin-dependent kinase inhibitors, including p21 and p27, and inducing extracellular matrix growth and fibroblast activity leading to tubulointerstitial fibrosis [58]. Such a hypothesis is supported by animal models demonstrating a lack of hypertrophy and enhanced hyperplasia in response to TGF-β or p21 knockout models [59, 60]. Moreover, such molecules are involved in the early senescence profile of PT cells in diabetic models.

### Tubular hyperreabsorption

PT alterations in the hyperglycaemic environment are not limited to hyperplasia and hypertrophy but also involve enhanced tubular reabsorption of various electrolytes and glucose [61, 62]. However, such physiological mechanisms are closely linked. Hyperglycaemia induces the upregulation of SGLT2 [43, 61], which prompts intensified glucose and sodium reabsorption via SGLT2 and SGLT1 in the PT, alongside heightened passive reabsorption of chloride and water [63]. Reduced sodium ion delivery to the juxtaglomerular apparatus leads to modulation to the renin–angiotensin II axis [64, 65]. Consequently, hyperreabsorption of sodium and chloride triggers tubule–glomerular feedback mechanisms, increasing the GFR [64]. Renal hyperfiltration imposes additional stress on tubular cells and elevates the oxygen demand for reabsorption processes [43, 63, 66]. SGLT1-dependent sodium–glucose co-transport on macula densa cells prompts nitric oxide production and contributes to glomerular hyperfiltration [67].

SGLT2 inhibition counteracts glomerular hyperfiltration by reducing sodium–glucose transport, restoring tubule–glomerular feedback signals and lowering the single-nephron GFR. SGLT2 inhibitors, by blocking glucose and sodium reabsorption, enhancing sodium delivery at the juxtaglomerular apparatus, triggering tubule–glomerular feedback, increasing adenosine, augmenting afferent arteriole tone, reducing glomerular hyperfiltration and lowering renal oxygen needs and neuroendocrine activation and decreasing albuminuria [30]. These mechanisms explain the initial decline followed by stabilization of the GFR and a reduction in CKD progression [63, 66]. Various mechanisms are involved in the beneficial effects of SGLT2 inhibitors regarding renal protection and mortality [68, 69]. In a preclinical study conducted on human kidney PT cell lines in a hyperglycaemic environment, exposure to empagliflozin over a 72-hour period led to attenuation of hyperglycaemia-mediated overexpression of interleukin-6 (IL-6), Toll-like receptor-4, type IV collagen, activator protein-1 and nuclear deoxyribonucleic acid binding for nuclear factor κB without a compensatory overexpression of neither SGLT1 or GLUT2 [70]. As pro-inflammatory and pro-fibrotic signalling pathways are central mechanisms of diabetic nephropathy and diabetes-associated PT hypertrophy, the effects of SGLT2 inhibitor therapy on such pathways are fundamental for understanding their role in PTs.

Empagliflozin therapy has been shown to downregulate transcription of genes encoding for various pro-inflammatory cytokines, including IL-1β, IL-6, monocyte chemoattractant protein-1 and tumour necrosis factor α, at macrophages [71]. Such anti-inflammatory effects of various SGLT2 inhibitors, including empagliflozin, dapagliflozin, canagliflozin and ipragliflozin, have been well-established in preclinical studies [72–74]. Moreover, SGLT2 inhibitor therapy may reduce mTOR activity by enhancing AMPK activity indirectly by altering the AMP–ATP balance in favour of AMP [75]. Moreover, dapagliflozin therapy has been shown to protect against diabetes-induced endothelial injury mediated via the AMPK/sirtuin 1 signalling pathway and reactive oxygen species [76]. Additional, anti-inflammatory beneficial effects of SGLT2 inhibitor therapy on PT structure and function includes a decrease in the M1:M2 macrophage ratio and NLRP3 inflammasome, a decrease in TGF-β levels and intracellular calcium levels [71]. Furthermore, a recent observational prospective cohort study involving 42 CKD patients who initiated SGLT2 inhibitor therapy demonstrated a significant decline in overhydration status that correlated with a decline in both albuminuria and glycosuria over the 6-month trial period [77]. Nevertheless, the small sample size, observational study design, lack of a control group and relatively short follow-up period are major limitations of this study.

The SGLT2 inhibitor luseogliflozin reduces cortical megalin expression, a protein involved in the endocytic handling of substances filtered by the glomeruli. This decrease in megalin expression was associated with decreased albumin uptake in the PTs. This study suggests that luseogliflozin may protect against tubular injury by inhibiting megalin expression, potentially reducing albuminuria [78]. This indicates that SGLT2 inhibition may alter oxygen metabolism in the PTs, potentially influencing their function. Inhibition of SGLT2 elicits multifaceted cardiorenal protective effects through various mechanisms. SGLT2 inhibitors induce osmotic diuresis and sodium excretion by impeding glucose reabsorption, consequently mitigating volume retention, hypertension and hyperuricaemia. Additionally, metabolic adaptations such as heightened lipolysis can occur, bolstering these protective actions [79]. Notably, the functional reduction of glomerular pressure and filtration rate by SGLT2 inhibition preserves tubular integrity and maintains GFR over the long term [79]. Furthermore, the ancillary effects of SGLT2 inhibitors on the heart and microbiome further fortify their cardiorenal protective profile [79].

## THE PATHOPHYSIOLOGICAL ROLE OF PTS IN OBESITY

The multifaceted interplay between PT function, renin–angiotensin–aldosterone system (RAAS) activation and obesity-related glomerulopathy is illustrated in Fig. 2. Due to the heightened metabolic and haemodynamic demands associated with obesity, PT undergoes hypertrophy in response to the need to increase sodium, water and albumin absorption imposed by hyperfiltration [80, 81]. In this milieu, SGLT2 expression is also notably enhanced [52, 81, 82]. The RAAS, which is upregulated in obesity, contributes to glomerular hyperfiltration [83]. Angiotensin II and aldosterone increase the transcapillary hydraulic pressure difference at the glomerular level by contracting efferent arterioles more than afferent arterioles. Additionally, angiotensin II stimulates sodium absorption and activates epithelial sodium channels, leading to increased sodium reabsorption in the proximal and distal tubules [83]. Aldosterone, elevated in obesity, generates reactive oxygen species that damage podocytes and contribute to kidney damage [83, 84].

Glomerulotubular balance (GTB) and tubule–glomerular feedback ensure that a consistent fraction of the filtered load is reabsorbed across varying GFRs, primarily operating in the PTs, where ≈70% of sodium and water reabsorption occurs irrespective of GFR levels [85]. The phenomenon of tubular overload due to glomerular hyperfiltration may prompt increased sodium and water reabsorption in the PTs via GTB. Tubule–glomerular feedback, on the other hand, regulates preglomerular vascular resistance to maintain glomerular blood flow and GFR [85].

A retrospective observational study investigated renal alterations in obese individuals with proteinuria and glomerular hyperfiltration, focusing on PT hypertrophy and enlarged glomerular and tubular urinary spaces [86]. Through analysis of kidney biopsies from obese and lean subjects, the study showed that obese individuals display significantly increased volumes of the glomerular tuft and Bowman's space, along with hypertrophy of the PT epithelium and expanded tubular lumen [86]. Notably, the number of nuclei per PT profile was similar across groups, indicating hypertrophy as the primary mechanism [86]. Another study [87] showed a positive correlation between body mass index (BMI) and triglyceride content in the kidney cortex, suggesting a propensity for lipid accumulation in individuals with a higher BMI [87]. Lipid droplets were predominantly observed in PT cells, underscoring their association with obesity-related changes in the kidney [87]. These findings in humans align with observations in animal models of diet-induced obesity that demonstrated increased lipid accumulation in several parts of the renal structures [88].

In mice subjected to a high-fat diet (HFD) for 3 weeks, PT hypertrophy and damage ensued, albeit without a concurrent increase in albuminuria [89]. However, extending the HFD to 12 weeks induced PT hypertrophy and damage and elevated albuminuria, accompanied by RhoA and its effector Rho-associated protein kinase 1 activation, but no increase in protein diaphanous homolog (mDIA1) levels [89]. The mechanism underlying PT hypertrophy involved cell cycle arrest and the downregulation of p27Kip1, a multifunctional cyclin-dependent kinase inhibitor, due to RhoA activation [89]. A significant correlation between PT cell size and BMI, PT cell damage and mDIA1 expression was documented in this study. In another *in vivo* study [90], the size of PT cells was increased in mice on the HFD regimen compared with those on a low-fat diet (LFD), in addition to pronounced mesangial hypercellularity and enlarged glomerular on histological analysis [90]. PHD2 knockout mice, i.e. a model with the PHED2 gene knocked out (PHD2, also known as prolyl hydroxylase domain 2, regulates the stability of hypoxia-inducible factor), showed increased peritubular capillaries and enhanced expression of hypoxia-responsive genes compared with control HFD. These findings highlight the relevance of aberrant hypoxic responses due to PHD2 dysfunction in obesity-induced kidney injury and indicate that the hypertrophic PT cells observed in obese individuals have inadequate oxygen delivery, potentially leading to a state of hypoxia [90]. In obesity-induced kidney injury, tubular hypertrophy could arise from an insufficient hypoxic response within the PTs. Similarly, mice exposed to an HFD and obesity exhibited lipid accumulation in both glomeruli and PTs, albuminuria, increased systolic blood pressure, oxidative stress and a larger glomerular tuft area and mesangial matrix size compared with counterparts on an LFD [91].

Considering the effectiveness of SGLT2 inhibitors in managing CKD in diabetic and non-diabetic individuals and the increased SGLT2 expression in obesity [92], studying the potential of SGLT2 inhibitors in managing obesity-related renal damage and obesity-associated PT modifications is a promising research area.

## UNDERSTANDING PT HYPERTROPHY AND HYPERFUNCTION IN HF AND SUDDEN CARDIAC DEATH (SCD)

The favourable cardiovascular outcomes observed in HF patients with reduced and preserved ejection fraction by SGLT2 inhibitors in a recent systematic review [93] highlight the therapeutic potential of focusing on PT function in HF management. RAAS and sympathetic overactivation enhance Na^+^ and water reabsorption in the PTs in HF [94]. Secondary to enhanced proximal sodium reabsorption, the sodium concentration in the macula densa decreases, activating a tubule–glomerular reflex that causes dilatation of the afferent arteriole and glomerular hypertension, which increases the filtration fraction and helps maintain the GFR. The elevated filtration fraction increases peritubular capillary oncotic pressure, augmenting water and sodium reabsorption. Moreover, renal venous hypertension, a feature of decompensated HF, opposes hydrostatic pressure at the glomerular level and slows urine flow across the tubule, favouring sodium hyperreabsorption [94]. In addition, increased renal lymph flow in HF reduces colloid osmotic pressure in the renal interstitium, promoting passive Na^+^ reabsorption. Less sodium is delivered to the distal parts of the nephron, which impairs the efficacy of diuretics and endogenous natriuretic peptides. As alluded to before, the increased fractional sodium and chloride reabsorption in the PTs decreases the delivery of these ions to the macula densa, further activating neurohumoral pathways and contributing to disease progression in HF. Drugs targeting the PTs, like acetazolamide [95] and SGLT2 inhibitors [96], counteract these mechanisms and restore sodium excretion.

Reduced tubular maximum phosphate reabsorption capacity (TmP/GFR), a marker of PT function, is associated with the severity of HF and evidence of tubular dysfunction and/or damage as indicated by increased neutrophil gelatinase-associated lipocalin (NGAL) excretion [97]. Reduced TmP/GFR independently predicted an increased risk of plasma NGAL doubling, i.e. tubular function worsening, and adverse clinical outcomes, including all-cause mortality and HF hospitalization [97]. Remarkably, SGLT2 inhibition by empagliflozin enhanced TmP/GFR in patients with acute HF, highlighting the importance of PT function in HF pathophysiology.

A preclinical investigation in wild-type and inducible IGF-1 receptor knockout mice [98] showed that furosemide triggers hypertrophic changes in PT after 3 weeks of treatment. After an initial hyperplastic phase, distal tubular segments also developed hypertrophy in these models [98]. The study identified IGF-1 receptor (IGF-1R) signalling as a key mechanism mediating these effects. Additionally, the upregulation of IGF-1 binding protein 2 in the PTs of furosemide-treated mice pointed to the specificity of IGF-1R-dependent responses in PT remodelling [98].

Meta-analyses of clinical trials underscore the potential impact of SGLT2 inhibitors on SCD and cardiac arrhythmias, including ventricular arrhythmias (VAs) across various patient categories, including HF, diabetes and CKD [99–101]. The ability of SGLT2 inhibitors to directly and indirectly reduce NHE3 activity and suppress late sodium currents likely contributes to their anti-arrhythmic properties [96, 102]. Canagliflozin therapy has been shown to inhibit PT NHE3 activity independent of glucose levels [103]. These findings triggered speculations on the potential role of PTs in modulating cardiac electrophysiology and arrhythmogenesis [102]. This possibility demands specific experimental studies in animal models and well-designed mechanistic studies in patients with heart disease.

## THERAPEUTIC APPROACHES AIMED AT PTS

**Figure 3:**
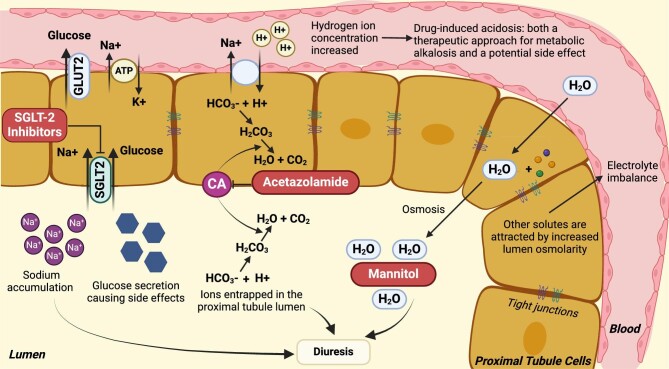
Therapeutic agents targeting PTs and their mechanism of actions. H_2_O: water.

Osmotic diuretics, including mannitol, adenylate cyclase inhibitors (e.g. methylxanthines) and carbonic anhydrase inhibitors (e.g. acetazolamide), impact proximal sodium reabsorption (Fig. 3, Table 2) [104]. Neither adenylate cyclase inhibitors nor osmotic diuretics showed meaningful efficacy for treating cardiorenal disorders. As previously alluded to, acetazolamide has recently been shown to be an effective measure for patients with decompensated, congestive HF. In a multicentre double-blind randomized placebo-controlled clinical trial in 519 patients with acute decompensated HF, acetazolamide (500 mg/day intravenously) for 3 days led to higher rates of successful decongestion (42.2% versus 30.5%; *P* < .001) and higher urine output without adverse effects on renal function or electrolytes [105]. This effect was independent of CKD stage and did not impact kidney function [95]. These findings have been confirmed in a meta-analysis involving 559 patients from three clinical studies [106]. Acetazolamide tolerance and the potential risk for metabolic acidosis in response to PT bicarbonaturia demands that this drug be administered no more than 3–4 days/week [107].

**Table 2:** List of the drugs that are transported from PTs and associated transporters.

Filtered drugs	Drug class	Filtration transporters
Metformin	Antidiabetic	OCT2
Cisplatin	Antineoplastic	OCT2
Methotrexate	Antineoplastic	OAT1/OAT3
6-Fluoruracil	Antineoplastic	OAT2
Taxol	Antineoplastic	OAT2
Sartanes	Angiotensin II receptor blockers	OAT1/OAT3
Chlorothiazide/cyclothiazide	Thiazide diuretic	OAT1
Acetolazamide/methazolamide	Carbonic anhydrase inhibitors	OAT1
Furosemide/bumetanide	Loop diuretics	OAT1/OAT3
Cefazolin/cephalexin	Cephalosporins	OAT1/OAT3
Acyclovir	Antiviral	OAT1/OAT2
Adefovir	Antiviral	OAT1/OAT3
Zidovudine	Antiviral	OAT1/OAT3
Valocyclovir	Antiviral	OAT3
Didanosine	Antiviral	OAT3
Tenofovir	Antiviral	OAT1/OAT3
Cimetidine	H2 receptor antagonist	OAT1/OAT3
Ranitidine	H2 receptor antagonist	OAT1/3
Famotidine	H2 receptor antagonist	OAT3
Statins	HMG CoA reductase inhibitor	OAT3
Benzylpenicilin	Antibiotics	OAT3
Tetracycline	Antibiotics	OAT3
Indomethacin	NSAID	OAT3
Ketoprofen	NSAID	OAT3

NSAID: Non-steroidal anti-inflammatory drug; OCT: organic cation transporter.

SGLT2 inhibitors are game-changer therapeutic alternatives in the management of type 2 DM and congestive HF. SGLT2 inhibitors led to the phosphorylation of the apical NHE3 channel, a PT antiporter involved in reabsorbing approximately two-thirds of filtered sodium [108]. SGLT2 inhibitors are also involved in inhibiting sodium–phosphate co-transporter 2a, a PT transporter involved in phosphate reabsorption, resulting in hyperphosphataemia and elevated levels of fibroblast growth factor-23 and PTH [109, 110], which might worsen mineral and bone disorder in CKD patients. SGLT2 inhibitor therapy increases the fractional excretion of urate through unclear pathophysiological mechanisms [111]. The net effect of SGLT2 inhibitors on the RAAS or sympathetic activity, two compensatory mechanisms leading to worsening cardiorenal outcomes during more extended follow-up periods, is unclear. A study in nine patients with type 2 DM receiving various SGLT2 inhibitor agents showed a decline in total urinary angiotensinogen:creatinine ratio and intact urinary angiotensinogen:creatinine ratio, suggesting a downregulation of RAAS during prolonged treatment with these drugs [112]. In contrast, a clinical trial of 40 type 1 DM patients treated with empagliflozin 25 mg/day documented a considerable increase in aldosterone and angiotensin II serum levels and a decline in plasma nitric oxide levels [113]. The underlying pathophysiologic mechanisms point to NHE3 inhibition, the crucial renal mechanism of SGLT2 inhibitors, in reducing RAAS activation via increased sodium concentration in the macula densa in the first study [103] and to volume contraction–mediated upregulation of renin and SGLT2 expression in the second study [113]. The prevailing view is that the SGLT2 inhibitor diuretic effect is observed in the early phase of treatment, which activates the systemic RAAS, leading to an initial increase of renin levels in the first 3 months of therapy [114]. Conversely, the effects on volume changes are attenuated in chronically treated patients. A new steady state is reached due to a counterregulatory effect, resulting in lower body sodium concentration and blood volume [114]. Furthermore, attenuating hyperglycaemia and angiotensin II in response to SGLT2 inhibitor therapy may improve PT inflammation and oxidative stress [70]. Indeed, dapagliflozin downregulates renal inflammatory gene expression in a mice model of type 1 DM [115].

The mechanism and the outcome behind hyperuricaemia as an actor for nephron damage, hypertrophy/hyperfiltration and glomerulosclerosis are similar to that of hyperglycaemia [116]. Therefore, we hypothesized that the use of URAT1 inhibitors may be as functional as SGLT2 inhibitors. In hyperuricaemic patients, after the body filters the high uric acid in the blood through the Bowman capsule, it is reabsorbed up to 90% by URAT1 in the proximal convoluted tubule [117]. The high amount of uric acid in the nephron puts an excessive load on the URAT1 transporter. Consequently, the kidney undergoes hypertrophy or hyperfunctioning to meet the need for 90% reabsorption of uric acid, as happens in nearly all conditions that cause the nephrons to overwork. Besides increased workload, the metabolic effect of high uric acid levels also causes problematic outcomes [118]. This stress and compensation mechanism eventually reaches an unbearable level, leading to nephron sclerosis or another form of non-functional state. To prevent these conditions and minimize their effects, URAT1 inhibitors can be used. With the use of URAT1 inhibitors, the high amount of uric acid in the nephron will not be reabsorbed by URAT1 in the PT and will be excreted in the urine [119]. In this way, the nephron will not try to transport as much uric acid as it can and will be minimally affected by the stress and growth-inducing factors that increase while trying to transport the high amount of uric acid. Consequently, damage to the kidney and nephrons will be minimized. Additionally, negative effects on the body due to hypofunctioning kidneys will be decreased. However, the use of URAT1 inhibitors is controversial because of the high side-effect profile [120]. Therefore, risk–benefit studies of the use of URAT1 inhibitors for the prevention or retarding the progression of nephropathy in a hyperuricaemic state and the development of and research on novel drugs should be encouraged.

Another aspect to consider while evaluating the role of PTs is drug delivery and uptake to PT cells. Initial barriers include negatively charged glomerular endothelial cells, glomerular basement membrane containing laminins, type IV collagen heparan sulphate and podocytes. After passing these barriers, drugs are taken up by PT cells either via transporter proteins or receptor-mediated endocytosis [121]. Solute carrier and ATP-binding cassette transporters are relatively ineffective in transporting large molecules [122, 123]. Receptor-mediated endocytosis is the primary mechanism mediating drug delivery and includes four major proteins: folate receptors, megalin, cubilin and amnionless [124]. The S1 segment of the PT is the primary site for receptor-mediated endocytosis [125]. Megalin and cubilin cooperate in the transport of nearly all plasma proteins. Drug delivery is a significant challenge in drug discovery, particularly when developing treatments that target the PTs of the kidneys. Due to their location and the complex physiological barriers present, delivering drugs precisely to these structures can be difficult. In response to these challenges, various strategies to improve drug delivery to the PTs are being explored. These include nanoparticle carriers, prodrugs that become activated specifically within the PTs, ligand-directed delivery (i.e. drugs conjugated with ligands that specifically bind to receptors or antigens expressed on the surface of PT cells), gene therapy and local delivery system (i.e. catheter-based delivery systems whereby drugs are directly conveyed to the kidney via the renal arteries). Continued research and development in these areas are crucial for overcoming the challenges associated with drug delivery to the PTs and for advancing new treatments for kidney-related diseases.

## LIMITATIONS

PT hyperplasia, hypertrophy and hyperfunctioning are rapidly growing pathological concepts primarily reported in diabetic and/or obese patients. Nevertheless, there is currently no consensus on the ideal histopathological method to assess tubular hyperplasia or hypertrophy or clear cut-off values for such conditions. Moreover, current literature regarding such issues primarily depends upon preclinical studies conducted in animal models or human cell lines with little to no knowledge from clinical studies. Therefore, potential applicability of such data to the general population or kidney diseases is unclear. Along with the lack of established criteria for tubular hyperplasia and hypertrophy, it is unclear whether beneficial effects of PT-targeting therapies may be attributable to their effects on PT structure despite their hypothetical role in reversing or preventing PT structural alterations. As such, multiple PT-targeting pharmacotherapeutic approaches have additional roles at glomeruli, distal tubules and non-renal structures that may potentially complicate the evaluation of their beneficial role on kidney health.

## CONCLUSION

The research surrounding the physiological role of PTs and the impact of pathological events on these structures, leading to various disease states, is rapidly growing. Multiple pharmacotherapeutic options are being explored to address these issues. Despite the increasing understanding of PTs and ongoing preclinical and clinical studies focusing on pathophysiology and treatment, there is still a vast amount to be discovered in this novel area of research. While there are currently only a limited number of trials specifically targeting PTs, one notable clinical trial (NCT05998837) is examining alterations in urinary PT epithelial cells in CKD patients, both with and without diabetes, who are being treated with dapagliflozin compared with placebo. This study aims to investigate various markers of kidney senescence, inflammation and damage in these patients. It is crucial to acknowledge the significance of PT hypertrophy and hyperfunctioning in the pathophysiology of various disorders, including CKD, congestive heart failure, DM and metabolic syndrome. Understanding the role of PT in these conditions can provide valuable insights for developing effective treatment strategies.

## Data Availability

All data are available in the article.

## References

[1] Lemley KV, Kriz W. Anatomy of the renal interstitium. Kidney Int 1991;39:370–81. 10.1038/ki.1991.49 2062030

[2] Greger R . Physiology of renal sodium transport. Am J Med Sci 2000;319:51–62. 10.1016/S0002-9629(15)40679-2 10653444

[3] Ghezzi C, Loo DDF, Wright EM. Physiology of renal glucose handling via SGLT1, SGLT2 and GLUT2. Diabetologia 2018;61:2087–97. 10.1007/s00125-018-4656-5 30132032 PMC6133168

[4] Scheepers A, Joost HG, Schurmann A. The glucose transporter families SGLT and GLUT: molecular basis of normal and aberrant function. JPEN J Parenter Enteral Nutr 2004;28:364–71. 10.1177/0148607104028005364 15449578

[5] Chevalier RL . The proximal tubule is the primary target of injury and progression of kidney disease: role of the glomerulotubular junction. Am J Physiol Renal Physiol 2016;311:F145–61. 10.1152/ajprenal.00164.2016 27194714 PMC4967168

[6] Hall AM, Trepiccione F, Unwin RJ. Drug toxicity in the proximal tubule: new models, methods and mechanisms. Pediatr Nephrol 2022;37:973–82. 10.1007/s00467-021-05121-9 34050397 PMC9023418

[7] Payne RB . Renal tubular reabsorption of phosphate (TmP/GFR): indications and interpretation. Ann Clin Biochem 1998;35:201–6. 10.1177/000456329803500203 9547891

[8] Lavecchia AM, Pelekanos K, Mavelli F et al. Cell hypertrophy: a “biophysical roadblock” to reversing kidney injury. Front Cell Dev Biol 2022;10:854998. 10.3389/fcell.2022.854998 35309910 PMC8927721

[9] Impact of diabetes on the effects of sodium glucose co-transporter-2 inhibitors on kidney outcomes: collaborative meta-analysis of large placebo-controlled trials. Lancet 2022;400:1788–801. 10.1016/S0140-6736(22)02074-8 36351458 PMC7613836

[10] Chrysopoulou M, Rinschen MM. Metabolic rewiring and communication: an integrative view of kidney proximal tubule function. Annu Rev Physiol 2024;86:405–27. 10.1146/annurev-physiol-042222-024724 38012048

[11] Clark JZ, Chen L, Chou CL et al. Representation and relative abundance of cell-type selective markers in whole-kidney RNA-seq data. Kidney Int 2019;95:787–96. 10.1016/j.kint.2018.11.028 30826016 PMC7466803

[12] Wilcox CS . Antihypertensive and renal mechanisms of SGLT2 (sodium-glucose linked transporter 2) inhibitors. Hypertension 2020;75:894–901. 10.1161/HYPERTENSIONAHA.119.11684 32114848

[13] Wright EM . Renal Na^+^-glucose cotransporters. Am J Physiol Renal Physiol 2001;280:F10–8. 10.1152/ajprenal.2001.280.1.F10 11133510

[14] Gonska T, Hirsch JR, Schlatter E. Amino acid transport in the renal proximal tubule. Amino Acids 2000;19:395–407. 10.1007/s007260070019 11128548

[15] Verrey F, Singer D, Ramadan T et al. Kidney amino acid transport. Pflugers Arch 2009;458:53–60. 10.1007/s00424-009-0638-2 19184091

[16] Christensen EI, Gburek J. Protein reabsorption in renal proximal tubule-function and dysfunction in kidney pathophysiology. Pediatr Nephrol 2004;19:714–21. 10.1007/s00467-004-1494-0 15146321

[17] De S, Kuwahara S, Saito A. The endocytic receptor megalin and its associated proteins in proximal tubule epithelial cells. Membranes 2014;4:333–55. 10.3390/membranes4030333 25019425 PMC4194038

[18] Fromm M, Piontek J, Rosenthal R et al. Tight junctions of the proximal tubule and their channel proteins. Pflugers Arch 2017;469:877–87. 10.1007/s00424-017-2001-3 28600680

[19] Wang X, Armando I, Upadhyay K et al. The regulation of proximal tubular salt transport in hypertension: an update. Curr Opin Nephrol Hypertens 2009;18:412–20. 10.1097/MNH.0b013e32832f5775 19654544 PMC3722593

[20] Fuchs MA, Wolf M. Renal proximal tubule cells: power and finesse. J Clin Invest 2023;133:e169607. 10.1172/JCI169607 37115697 PMC10145182

[21] Alexander RT, Dimke H. Effects of parathyroid hormone on renal tubular calcium and phosphate handling. Acta Physiol (Oxf) 2023;238:e13959. 10.1111/apha.13959 36894509

[22] Eguchi S, Kawai T, Scalia R et al. Understanding angiotensin II type 1 receptor signaling in vascular pathophysiology. Hypertension 2018;71:804–10. 10.1161/HYPERTENSIONAHA.118.10266 29581215 PMC5897153

[23] Cano A, Miller RT, Alpern RJ et al. Angiotensin II stimulation of Na-H antiporter activity is cAMP independent in OKP cells. Am J Physiol 1994;266:C1603–8. 10.1152/ajpcell.1994.266.6.C1603 8023891

[24] Carey RM, Wang ZQ, Siragy HM. Role of the angiotensin type 2 receptor in the regulation of blood pressure and renal function. Hypertension 2000;35:155–63. 10.1161/01.HYP.35.1.155 10642292

[25] Horita S, Nakamura M, Suzuki M et al. The role of renal proximal tubule transport in the regulation of blood pressure. Kidney Res Clin Pract 2017;36:12–21. 10.23876/j.krcp.2017.36.1.12 28428931 PMC5331971

[26] Nakamura M, Tsukada H, Seki G et al. Insulin promotes sodium transport but suppresses gluconeogenesis via distinct cellular pathways in human and rat renal proximal tubules. Kidney Int 2020;97:316–26. 10.1016/j.kint.2019.08.021 31735358

[27] Nigam SK, Bush KT, Martovetsky G et al. The organic anion transporter (OAT) family: a systems biology perspective. Physiol Rev 2015;95:83–123. 10.1152/physrev.00025.2013 25540139 PMC4281586

[28] Yu Z, Zhang J, Liang Z et al. Pancreatic hormone insulin modulates organic anion transporter 1 in the kidney: regulation via remote sensing and signaling network. AAPS J 2023;25:13. 10.1208/s12248-022-00778-y 36627500 PMC10695010

[29] Tang SC, Lai KN. The pathogenic role of the renal proximal tubular cell in diabetic nephropathy. Nephrol Dial Transplant 2012;27:3049–56. 10.1093/ndt/gfs260 22734110

[30] Vallon V, Thomson SC. Targeting renal glucose reabsorption to treat hyperglycaemia: the pleiotropic effects of SGLT2 inhibition. Diabetologia 2017;60:215–25. 10.1007/s00125-016-4157-3 27878313 PMC5884445

[31] Pollock CA, Bostrom TE, Dyne M et al. Tubular sodium handling and tubuloglomerular feedback in compensatory renal hypertrophy. Pflugers Arch 1992;420:159–66. 10.1007/BF00374985 1620575

[32] Fine LG, Bradley T. Adaptation of proximal tubular structure and function: insights into compensatory renal hypertrophy. Fed Proc 1985;44:2723–7. 2410299

[33] Wolf G, Neilson EG. Molecular mechanisms of tubulointerstitial hypertrophy and hyperplasia. Kidney Int 1991;39:401–20. 10.1038/ki.1991.52 1648145

[34] Uehara-Watanabe N, Okuno-Ozeki N, Minamida A et al. Direct evidence of proximal tubular proliferation in early diabetic nephropathy. Sci Rep 2022;12:778. 10.1038/s41598-022-04880-1 35039597 PMC8763925

[35] Wolf G, Neilson EG. Angiotensin II induces cellular hypertrophy in cultured murine proximal tubular cells. Am J Physiol 1990;259:F768–77. 1700629 10.1152/ajprenal.1990.259.5.F768

[36] Liu BC, Chen L, Sun J et al. Connective tissue growth factor-mediated angiotensin II-induced hypertrophy of proximal tubular cells. Nephron Exp Nephrol 2006;103:e16–26. 10.1159/000090504 16374037

[37] Liu B, Preisig PA. Compensatory renal hypertrophy is mediated by a cell cycle-dependent mechanism. Kidney Int 2002;62:1650–8. 10.1046/j.1523-1755.2002.00620.x 12371965

[38] Pollock CA, Field MJ. Compensatory renal hypertrophy: tubular cell growth and transport studied in primary culture. Nephron 1993;64:615–20. 10.1159/000187410 8366989

[39] Golchini K, Norman J, Bohman R et al. Induction of hypertrophy in cultured proximal tubule cells by extracellular NH4Cl. J Clin Invest 1989;84:1767–79. 10.1172/JCI114361 2480366 PMC304054

[40] Silva Dos Santos D, Polidoro JZ, Borges-Junior FA et al. Cardioprotection conferred by sodium-glucose cotransporter 2 inhibitors: a renal proximal tubule perspective. Am J Physiol Cell Physiol 2020;318:C328–36. 10.1152/ajpcell.00275.2019 31721613

[41] Nassif ME, Windsor SL, Borlaug BA et al. The SGLT2 inhibitor dapagliflozin in heart failure with preserved ejection fraction: a multicenter randomized trial. Nat Med 2021;27:1954–60. 10.1038/s41591-021-01536-x 34711976 PMC8604725

[42] Gilbert RE . Proximal tubulopathy: prime mover and key therapeutic target in diabetic kidney disease. Diabetes 2017;66:791–800. 10.2337/db16-0796 28325740

[43] Vallon V . The proximal tubule in the pathophysiology of the diabetic kidney. Am J Physiol Regul Integr Comp Physiol 2011;300:R1009–22. 10.1152/ajpregu.00809.2010 21228342 PMC3094037

[44] Vallon V, Thomson SC. Renal function in diabetic disease models: the tubular system in the pathophysiology of the diabetic kidney. Annu Rev Physiol 2012;74:351–75. 10.1146/annurev-physiol-020911-153333 22335797 PMC3807782

[45] Wolf G, Ziyadeh FN. Molecular mechanisms of diabetic renal hypertrophy. Kidney Int 1999;56:393–405. 10.1046/j.1523-1755.1999.00590.x 10432377

[46] Vallon V, Sharma K. Sodium-glucose transport: role in diabetes mellitus and potential clinical implications. Curr Opin Nephrol Hypertens 2010;19:425–31. 10.1097/MNH.0b013e32833bec06 20539226 PMC5886710

[47] Habib SL . Kidney atrophy vs hypertrophy in diabetes: which cells are involved? Cell Cycle 2018;17:1683–7. 10.1080/15384101.2018.1496744 29995580 PMC6133324

[48] Wright EM, Turk E. The sodium/glucose cotransport family SLC5. Pflugers Arch 2004;447:510–8. 10.1007/s00424-003-1202-0 12748858

[49] Santer R, Calado J. Familial renal glucosuria and SGLT2: from a mendelian trait to a therapeutic target. Clin J Am Soc Nephrol 2010;5:133–41. 10.2215/CJN.04010609 19965550

[50] Vestri S, Okamoto MM, de Freitas HS et al. Changes in sodium or glucose filtration rate modulate expression of glucose transporters in renal proximal tubular cells of rat. J Membr Biol 2001;182:105–12. 10.1007/s00232-001-0036-y 11447502

[51] Tabatabai NM, Sharma M, Blumenthal SS et al. Enhanced expressions of sodium-glucose cotransporters in the kidneys of diabetic Zucker rats. Diabetes Res Clin Pract 2009;83:e27–30. 10.1016/j.diabres.2008.11.003 19095325 PMC2652566

[52] Rahmoune H, Thompson PW, Ward JM et al. Glucose transporters in human renal proximal tubular cells isolated from the urine of patients with non-insulin-dependent diabetes. Diabetes 2005;54:3427–34. 10.2337/diabetes.54.12.3427 16306358

[53] Huang HC, Preisig PA. G1 kinases and transforming growth factor-β signaling are associated with a growth pattern switch in diabetes-induced renal growth. Kidney Int 2000;58:162–72. 10.1046/j.1523-1755.2000.00151.x 10886561

[54] Satriano J, Vallon V. Primary kidney growth and its consequences at the onset of diabetes mellitus. Amino Acids 2006;31:1–9. 10.1007/s00726-006-0326-x 16733619

[55] Clifford A, Morgan D, Yuspa SH et al. Role of ornithine decarboxylase in epidermal tumorigenesis. Cancer Res 1995;55:1680–6. 7712475

[56] Levine JH, Buse MG, Leaming AB et al. Effect of streptozotocin-induced diabetes on renal ornithine decarboxylase activity. Diabetes 1980;29:532–5. 10.2337/diab.29.7.532 6445842

[57] Pedersen SB, Flyvbjerg A, Grønbaek H et al. Increased ornithine decarboxylase activity in kidneys undergoing hypertrophy in experimental diabetes. Mol Cell Endocrinol 1992;86:67–72. 10.1016/0303-7207(92)90176-7 1511780

[58] Kamesaki H, Nishizawa K, Michaud GY et al. TGF-β1 induces the cyclin-dependent kinase inhibitor p27Kip1 mRNA and protein in murine B cells. J Immunol 1998;160:770–7. 10.4049/jimmunol.160.2.770 9551912

[59] Al-Douahji M, Brugarolas J, Brown PA et al. The cyclin kinase inhibitor p21WAF1/CIP1 is required for glomerular hypertrophy in experimental diabetic nephropathy. Kidney Int 1999;56:1691–9. 10.1046/j.1523-1755.1999.00728.x 10571777

[60] Chen S, Hoffman BB, Lee JS et al. Cultured tubule cells from TGF-beta1 null mice exhibit impaired hypertrophy and fibronectin expression in high glucose. Kidney Int 2004;65:1191–204. 10.1111/j.1523-1755.2004.00492.x 15086458

[61] Bank N, Aynedjian HS. Progressive increases in luminal glucose stimulate proximal sodium absorption in normal and diabetic rats. J Clin Invest 1990;86:309–16. 10.1172/JCI114700 2365820 PMC296722

[62] Vallon V, Richter K, Blantz RC et al. Glomerular hyperfiltration in experimental diabetes mellitus: potential role of tubular reabsorption. J Am Soc Nephrol 1999;10:2569–76. 10.1681/ASN.V10122569 10589696

[63] Iacoviello M . Metabolic regulation in the development of cardiovascular disease and heart failure. Int J Mol Sci 2023;24:8773. 10.3390/ijms24108773 37240119 PMC10218119

[64] Sparks MA, Crowley SD, Gurley SB et al. Classical renin-angiotensin system in kidney physiology. Compr Physiol 2014;4:1201–28. 10.1002/cphy.c130040 24944035 PMC4137912

[65] Tonneijck L, Muskiet MH, Smits MM et al. Glomerular hyperfiltration in diabetes: mechanisms, clinical significance, and treatment. J Am Soc Nephrol 2017;28:1023–39. 10.1681/ASN.2016060666 28143897 PMC5373460

[66] Gronda E, Vanoli E, Iacoviello M et al. The benefit of sodium-glucose co-transporter inhibition in heart failure: the role of the kidney. Int J Mol Sci 2022;23:11987. 10.3390/ijms231911987 36233288 PMC9569705

[67] Song P, Huang W, Onishi A et al. Knockout of Na^+^-glucose cotransporter SGLT1 mitigates diabetes-induced upregulation of nitric oxide synthase NOS1 in the macula densa and glomerular hyperfiltration. Am J Physiol Renal Physiol 2019;317:F207–17. 10.1152/ajprenal.00120.2019 31091127 PMC6692722

[68] Sarafidis P, Pella E, Kanbay M et al. SGLT-2 inhibitors and nephroprotection in patients with diabetic and non-diabetic chronic kidney disease. Curr Med Chem 2023;30:2039–60. 10.2174/0929867329666220825121304 36028970

[69] Kalay Z, Sahin OE, Copur S et al. SGLT-2 inhibitors in nephrotic-range proteinuria: emerging clinical evidence. Clin Kidney J 2023;16:52–60. 10.1093/ckj/sfac189 36726436 PMC9871839

[70] Panchapakesan U, Pegg K, Gross S et al. Effects of SGLT2 inhibition in human kidney proximal tubular cells—renoprotection in diabetic nephropathy? PLoS One 2013;8:e54442. 10.1371/journal.pone.0054442 23390498 PMC3563635

[71] Schönberger E, Mihaljević V, Steiner K et al. Immunomodulatory effects of SGLT2 inhibitors-targeting inflammation and oxidative stress in aging. Int J Environ Res Public Health 2023;20:6671. 10.3390/ijerph20176671 37681811 PMC10487537

[72] Lee JY, Lee M, Lee JY et al. Ipragliflozin, an SGLT2 inhibitor, ameliorates high-fat diet-induced metabolic changes by upregulating energy expenditure through activation of the AMPK/SIRT1 pathway. Diabetes Metab J 2021;45:921–32. 10.4093/dmj.2020.0187 33611885 PMC8640151

[73] Naznin F, Sakoda H, Okada T et al. Canagliflozin, a sodium glucose cotransporter 2 inhibitor, attenuates obesity-induced inflammation in the nodose ganglion, hypothalamus, and skeletal muscle of mice. Eur J Pharmacol 2017;794:37–44. 10.1016/j.ejphar.2016.11.028 27876617

[74] Lee SG, Lee SJ, Lee JJ et al. Anti-inflammatory effect for atherosclerosis progression by sodium-glucose cotransporter 2 (SGLT-2) inhibitor in a normoglycemic rabbit model. Korean Circ J 2020;50:443–57. 10.4070/kcj.2019.0296 32153145 PMC7098824

[75] La Grotta R, Frigé C, Matacchione G et al. Repurposing SGLT-2 inhibitors to target aging: available evidence and molecular mechanisms. Int J Mol Sci 2022;23:12325. 10.3390/ijms232012325 36293181 PMC9604287

[76] Faridvand Y, Kazemzadeh H, Vahedian V et al. Dapagliflozin attenuates high glucose-induced endothelial cell apoptosis and inflammation through AMPK/SIRT1 activation. Clin Exp Pharmacol Physiol 2022;49:643–51. 10.1111/1440-1681.13638 35274762

[77] Schork A, Eberbach ML, Bohnert BN et al. SGLT2 inhibitors decrease overhydration and proteasuria in patients with chronic kidney disease: a longitudinal observational study. Kidney Blood Press Res 2024;49:124–34. 38228104 10.1159/000535643 PMC10885839

[78] Takiyama Y, Sera T, Nakamura M et al. Impacts of diabetes and an SGLT2 inhibitor on the glomerular number and volume in db/db mice, as estimated by synchrotron radiation micro-CT at SPring-8. EBioMedicine 2018;36:329–46. 10.1016/j.ebiom.2018.09.048 30322799 PMC6197731

[79] Vallon V . How can inhibition of glucose and sodium transport in the early proximal tubule protect the cardiorenal system? Nephrol Dial Transplant 2024;gfae060. 10.1093/ndt/gfae060 38439675 PMC11427065

[80] Hall JE, do Carmo JM, da Silva AA et al. Obesity, kidney dysfunction and hypertension: mechanistic links. Nat Rev Nephrol 2019;15:367–85. 10.1038/s41581-019-0145-4 31015582 PMC7278043

[81] Tsuboi N, Okabayashi Y, Shimizu A et al. The renal pathology of obesity. Kidney Int Rep 2017;2:251–60. 10.1016/j.ekir.2017.01.007 29142961 PMC5678647

[82] Bautista R, Manning R, Martinez F et al. Angiotensin II-dependent increased expression of Na^+^-glucose cotransporter in hypertension. Am J Physiol Renal Physiol 2004;286:F127–33. 10.1152/ajprenal.00113.2003 14506074

[83] Jiang Z, Wang Y, Zhao X et al. Obesity and chronic kidney disease. Am J Physiol Endocrinol Metab 2023;324:E24–41. 10.1152/ajpendo.00179.2022 36383637

[84] Yang S, Cao C, Deng T et al. Obesity-related glomerulopathy: a latent change in obesity requiring more attention. Kidney Blood Press Res 2020;45:510–22. 10.1159/000507784 32498064

[85] Tsuboi N, Okabayashi Y. The renal pathology of obesity: structure-function correlations. Semin Nephrol 2021;41:296–306. 10.1016/j.semnephrol.2021.06.002 34715960

[86] Tobar A, Ori Y, Benchetrit S et al. Proximal tubular hypertrophy and enlarged glomerular and proximal tubular urinary space in obese subjects with proteinuria. PLoS One 2013;8:e75547. 10.1371/journal.pone.0075547 24086563 PMC3783420

[87] Bobulescu IA, Lotan Y, Zhang J et al. Triglycerides in the human kidney cortex: relationship with body size. PLoS One 2014;9:e101285. 10.1371/journal.pone.0101285 25170827 PMC4149342

[88] Foster MC, Hwang SJ, Porter SA et al. Fatty kidney, hypertension, and chronic kidney disease: the Framingham Heart Study. Hypertension 2011;58:784–90. 10.1161/HYPERTENSIONAHA.111.175315 21931075 PMC3204377

[89] Ida-Naitoh M, Tokuyama H, Futatsugi K et al. Proximal-tubule molecular relay from early protein diaphanous homolog 1 to late Rho-associated protein kinase 1 regulates kidney function in obesity-induced kidney damage. Kidney Int 2022;102:798–814. 10.1016/j.kint.2022.05.018 35716954

[90] Futatsugi K, Tokuyama H, Shibata S et al. Obesity-induced kidney injury is attenuated by amelioration of aberrant PHD2 activation in proximal tubules. Sci Rep 2016;6:36533. 10.1038/srep36533 27827416 PMC5101521

[91] Deji N, Kume S, Araki S et al. Structural and functional changes in the kidneys of high-fat diet-induced obese mice. Am J Physiol Renal Physiol 2009;296:F118–26. 10.1152/ajprenal.00110.2008 18971213

[92] Heerspink HJL, Stefánsson BV, Correa-Rotter R et al. Dapagliflozin in patients with chronic kidney disease. N Engl J Med 2020;383:1436–46. 10.1056/NEJMoa2024816 32970396

[93] Vaduganathan M, Docherty KF, Claggett BL et al. SGLT-2 inhibitors in patients with heart failure: a comprehensive meta-analysis of five randomised controlled trials. Lancet 2022;400:757–67. 10.1016/S0140-6736(22)01429-5 36041474

[94] Mullens W, Verbrugge FH, Nijst P et al. Renal sodium avidity in heart failure: from pathophysiology to treatment strategies. Eur Heart J 2017;38:1872–82. 10.1093/eurheartj/ehx035 28329085

[95] Meekers E, Dauw J, Martens P et al. Renal function and decongestion with acetazolamide in acute decompensated heart failure: the ADVOR trial. Eur Heart J 2023;44:3672–82. 10.1093/eurheartj/ehad557 37623428

[96] Packer M, Wilcox CS, Testani JM. Critical analysis of the effects of SGLT2 inhibitors on renal tubular sodium, water and chloride homeostasis and their role in influencing heart failure outcomes. Circulation 2023;148:354–72. 10.1161/CIRCULATIONAHA.123.064346 37486998 PMC10358443

[97] Emmens JE, de Borst MH, Boorsma EM et al. Assessment of proximal tubular function by tubular maximum phosphate reabsorption capacity in heart failure. Clin J Am Soc Nephrol 2022;17:228–39. 10.2215/CJN.03720321 35131929 PMC8823926

[98] Krauson AJ, Walczak EM, Nizar JM et al. Abstract 19603: Chronic loop diuretic treatment induces tubular remodeling via IGF-1R dependent and IGF-1R independent signaling. Circulation 2017;136(Suppl 1):A19603.

[99] Li HL, Lip GYH, Feng Q et al. Sodium-glucose cotransporter 2 inhibitors (SGLT2i) and cardiac arrhythmias: a systematic review and meta-analysis. Cardiovasc Diabetol 2021;20:100. 10.1186/s12933-021-01293-8 33962654 PMC8106208

[100] Fernandes GC, Fernandes A, Cardoso R et al. Association of SGLT2 inhibitors with arrhythmias and sudden cardiac death in patients with type 2 diabetes or heart failure: a meta-analysis of 34 randomized controlled trials. Heart Rhythm 2021;18:1098–105. 10.1016/j.hrthm.2021.03.028 33757845

[101] Oates CP, Santos-Gallego CG, Smith A et al. SGLT2 inhibitors reduce sudden cardiac death risk in heart failure: meta-analysis of randomized clinical trials. J Cardiovasc Electrophysiol 2023;34:1277–85. 10.1111/jce.15894 36950852

[102] Sato T, Kouzu H, Yano T et al. Potential favorable action of sodium-glucose cotransporter-2 inhibitors on sudden cardiac death: a brief overview. Front Cardiovasc Med 2023;10:1159953. 10.3389/fcvm.2023.1159953 37252114 PMC10214280

[103] Albalawy WN, Youm EB, Shipman KE et al. SGLT2-independent effects of canagliflozin on NHE3 and mitochondrial complex I activity inhibit proximal tubule fluid transport and albumin uptake. Am J Physiol Renal Physiol 2024;326:F1041–53. 10.1152/ajprenal.00005.2024 38660713 PMC11381006

[104] Materson BJ . Insights into intrarenal sites and mechanisms of action of diuretic agents. Am Heart J 1983;106:188–208. 10.1016/0002-8703(83)90117-5 6869201

[105] Mullens W, Dauw J, Martens P et al. Acetazolamide in acute decompensated heart failure with volume overload. N Engl J Med 2022;387:1185–95. 10.1056/NEJMoa2203094 36027559

[106] Malik BA, Nnodebe I, Fayaz A et al. Effect of acetazolamide as add-on diuretic therapy in patients with heart failure: a meta-analysis. Cureus 2023;15:e37792. 37213994 10.7759/cureus.37792 PMC10198661

[107] Wongboonsin J, Thongprayoon C, Bathini T et al. Acetazolamide therapy in patients with heart failure: a meta-analysis. J Clin Med 2019;8:349. 10.3390/jcm8030349 30871038 PMC6463174

[108] Dominguez Rieg JA, Xue J, Rieg T. Tubular effects of sodium-glucose cotransporter 2 inhibitors: intended and unintended consequences. Curr Opin Nephrol Hypertens 2020;29:523–30. 10.1097/MNH.0000000000000632 32701600 PMC8772383

[109] Blau JE, Bauman V, Conway EM et al. Canagliflozin triggers the FGF23/1,25-dihydroxyvitamin D/PTH axis in healthy volunteers in a randomized crossover study. JCI Insight 2018;3:e99123. 10.1172/jci.insight.99123 29669938 PMC5931122

[110] Skeith MD, Healey LA, Cutler RE. Effect of phloridzin on uric acid excretion in man. Am J Physiol 1970;219:1080–2. 10.1152/ajplegacy.1970.219.4.1080 5459472

[111] Bailey CJ, Gross JL, Pieters A et al. Effect of dapagliflozin in patients with type 2 diabetes who have inadequate glycaemic control with metformin: a randomised, double-blind, placebo-controlled trial. Lancet 2010;375:2223–33. 10.1016/S0140-6736(10)60407-2 20609968

[112] Yoshimoto T, Furuki T, Kobori H et al. Effects of sodium-glucose cotransporter 2 inhibitors on urinary excretion of intact and total angiotensinogen in patients with type 2 diabetes. J Investig Med 2017;65:1057–61. 10.1136/jim-2017-000445 PMC5812257 28596160

[113] Cherney DZ, Perkins BA, Soleymanlou N et al. Renal hemodynamic effect of sodium-glucose cotransporter 2 inhibition in patients with type 1 diabetes mellitus. Circulation 2014;129:587–97. 10.1161/CIRCULATIONAHA.113.005081 24334175

[114] Sarzani R, Giulietti F, Di Pentima C et al. Sodium-glucose co-transporter-2 inhibitors: peculiar “hybrid” diuretics that protect from target organ damage and cardiovascular events. Nutr Metab Cardiovasc Dis 2020;30:1622–32. 10.1016/j.numecd.2020.05.030 32631704

[115] Hatanaka T, Ogawa D, Tachibana H et al. Inhibition of SGLT2 alleviates diabetic nephropathy by suppressing high glucose-induced oxidative stress in type 1 diabetic mice. Pharmacol Res Perspect 2016;4:e00239. 10.1002/prp2.239 28116093 PMC5242174

[116] Mazzali M, Kanellis J, Han L et al. Hyperuricemia induces a primary renal arteriolopathy in rats by a blood pressure-independent mechanism. Am J Physiol Renal Physiol 2002;282:F991–7. 10.1152/ajprenal.00283.2001 11997315

[117] Roch-Ramel F, Guisan B. Renal transport of urate in humans. News Physiol Sci 1999;14:80–4. 11390825 10.1152/physiologyonline.1999.14.2.80

[118] Kang DH, Nakagawa T, Feng L et al. A role for uric acid in the progression of renal disease. J Am Soc Nephrol 2002;13:2888–97. 10.1097/01.ASN.0000034910.58454.FD 12444207

[119] Leander J, Sunnåker M, Rekić D et al. A semi-mechanistic exposure-response model to assess the effects of verinurad, a potent URAT1 inhibitor, on serum and urine uric acid in patients with hyperuricemia-associated diseases. J Pharmacokinet Pharmacodyn 2021;48:525–41. 10.1007/s10928-021-09747-y 33728547 PMC8225519

[120] Chen Y, You R, Wang K et al. Recent updates of natural and synthetic URAT1 inhibitors and novel screening methods. Evid based Complement Alternat Med 2021;2021:5738900. 34754317 10.1155/2021/5738900 PMC8572588

[121] Ivanyuk A, Livio F, Biollaz J et al. Renal drug transporters and drug interactions. Clin Pharmacokinet 2017;56:825–92. 10.1007/s40262-017-0506-8. 28210973

[122] Nigam SK . The SLC22 transporter Family: a paradigm for the impact of drug transporters on metabolic pathways, signaling, and disease. Annu Rev Pharmacol Toxicol 2018;58:663–87. 10.1146/annurev-pharmtox-010617-052713 29309257 PMC6225997

[123] El-Sheikh AA, Masereeuw R, Russel FG. Mechanisms of renal anionic drug transport. Eur J Pharmacol 2008;585:245–55. 10.1016/j.ejphar.2008.02.085 18417112

[124] Christensen EI, Birn H, Storm T et al. Endocytic receptors in the renal proximal tubule. Physiology (Bethesda) 2012;27:223–36. 22875453 10.1152/physiol.00022.2012

[125] Schuh CD, Polesel M, Platonova E et al. Combined structural and functional imaging of the kidney reveals major axial differences in proximal tubule endocytosis. J Am Soc Nephrol 2018;29:2696–712. 10.1681/ASN.2018050522 30301861 PMC6218873

